# Risk mapping for better governance in biobanking: the case of biobank.cy

**DOI:** 10.3389/fgene.2024.1397156

**Published:** 2024-06-14

**Authors:** Kaya Akyüz, Melanie Goisauf, Gillian M. Martin, Michaela Th. Mayrhofer, Stella Antoniou, Georgia Charalambidou, Constantinos Deltas, Apostolos Malatras, Gregory Papagregoriou, Charalambos Stefanou, Mariel Voutounou

**Affiliations:** ^1^ Department of ELSI Services and Research, BBMRI-ERIC, Graz, Austria; ^2^ Department of Sociology, University of Malta, Msida, Malta; ^3^ Biobank.cy Center of Excellence in Biobanking and Biomedical Research, Nicosia, Cyprus; ^4^ University of Cyprus Medical School, Nicosia, Cyprus

**Keywords:** biobank, risk assessment, risk governance, research infrastructure, genetics, ELSI, risk mapping

## Abstract

**Introduction:** Risk governance is central for the successful and ethical operation of biobanks and the continued social license for being custodians of samples and data. Risks in biobanking are often framed as risks for participants, whereas the biobank’s risks are often considered as technical ones. Risk governance relies on identifying, assessing, mitigating and communicating all risks based on technical and standardized procedures. However, within such processes, biobank staff are often involved tangentially. In this study, the aim has been to conduct a risk mapping exercise bringing biobank staff as key actors into the process, making better sense of emerging structure of biobanks.

**Methods:** Based on the qualitative research method of situational analysis as well as the card-based discussion and stakeholder engagement processes, risk mapping was conducted at the biobank setting as an interactive engagement exercise. The analyzed material comprises mainly of moderated group discussions.

**Results:** The findings from the risk mapping activity are framed through an organismic metaphor: the biobank as a *growing*, *living* organism *in a changing environment*, where trust and sustainability are cross-cutting elements in making sense of the risks. Focusing on the situatedness of the dynamics within biobanking activity highlights the importance of prioritizing relations at the core of risk governance and promoting ethicality in the biobanking process by expanding the repertoire of considered risks.

**Conclusion:** With the organismic metaphor, the research brings the diverse group of biobank staff to the central stage for risk governance, highlighting how accounting for such diversity and interdependencies at the biobank setting is a prerequisite for an adaptive risk governance.

## 1 Introduction

Biological samples and data collected, stored and used in organized systems are becoming increasingly central to an internationalized, biomedical research landscape. In many parts of the globe, infrastructures, often under the umbrella term “biobanks,” are being built around biological samples and data, especially to foster research, and with this expansion, locating more than a thousand such biobanks has even become a task of its own as the emergence of biobank directories or locators signifies (O'Donoghue et al., 2021). Biobank as a concept has gone through a transformation from being merely a collection of samples and associated data in academic or hospital settings, to large-scale national repositories in the early 2000s (Cambon-Thomsen, 2004) to data-intensive, collaborative, international hubs for research in the last 20 years (Mayrhofer, 2013; Tupasela, 2021), especially in genomics. However, a universally agreeable definition is lacking (Mayrhofer, 2013), especially considering the diverse taxonomy including forms of public and commercial infrastructures as varied as disease-specific, population-based, national biobanks, along with the biospecimen/biodata foci, ranging from tissues to genomic data, blood and urine to demographic and health data (De Souza and Greenspan, 2013). Currently ten largest biobanks are in Austria, Canada, China, Finland, France, Qatar, the United Kingdom and the United States (Kinkorová, 2021), which shows that despite predominance of Europe and North America, there is much diversity in the biobanking landscape in terms of geography. With increasing variety in samples and data and the need to bring together such resources from different settings for research projects, biobanks are becoming commonplace in biomedicine where tensions around governance are attracting scholarly attention (Kaye, 2011; Lazareva et al., 2022). Considering the transborder movement of biological samples and data for research as well as long-term storage for varying and unknown purposes of use, with the associated opportunities for research and innovation, challenges and complexities emerge. These range from ethical, legal and societal issues, often called ELSI (Harris et al., 2012; Goisauf et al., 2019; Akyüz et al., 2021), to biobank sustainability (Simeon-Dubach and Watson, 2014; Watson et al., 2014; Chalmers et al., 2016). Not only is the academic literature enriched with continuous work on a wide range of ELSI topics, but practitioners also seek their own solutions to these (e.g., models for sustainability: https://www.bbmri.nl/services/knowledge/sustainable-biobanking).

As an infrastructure continuously in-the-making, the relevant legal and regulatory frameworks often do not directly focus on biobanking and are fragmented. While there are international rules (Council of Europe, 1997; UNESCO, 1998; Council of Europe, 2005; UNESCO, 2006; World Medical Association, 2013 [1964]; Council of Europe, 2016; World Medical Association, 2016), guidelines (e.g., OECD, 2009; Council for International Organizations of Medical Sciences, 2016; Astrin et al., 2018), legal frameworks (e.g., European Parliament and Council of the European Union, 2016) that are relevant, few countries have current biobanking laws (Kaye et al., 2016). Furthermore, the ways that these documents or tools translate into practice differ widely - from changing consent practices (Prictor et al., 2018; Mamo et al., 2020; Teare et al., 2021) to different ways of engaging participants (Kaye et al., 2012; Goisauf and Durnová, 2018; Haas et al., 2021). To navigate this terrain, scholars have tried developing checklists, toolkits and other forms of instruments that can aid in practice or provide an overview against a fragmented domain (Tzortzatou et al., 2021; Tzortzatou-Nanopoulou et al., 2023); however, it is clear one size fits all solutions are often not possible in this domain, not least due to lack of regulation, but rather due to the diversity within biobanking as well as across sites.

Focusing on publicly funded research-focused biobanks, these can be categorized into two although it is hard to agree on how to categorize such diverse and multi-functional institutions (Parodi, 2015): population-based biobanks (e.g., *Danish National Biobank*, *UK Biobank* or *biobank.cy*) and disease oriented/condition-focused biobanks (e.g., *The International Agency for Research on Cancer Biobank*, *MRC Centre Neuromuscular Biobank* or *The Norwegian Childhood Diabetes Registry and Biobank*) while many biobanks serve both purposes. The wider aim of publicly funded biobanks is to provide samples and associated data for different types of studies or research to improve human health. As infrastructures that rely on continuous support of the publics, not only financially, but also with contributions of bodily samples and data for specific research and also longer term uses, biobanks need to have good governance models in place that ensure risks are rightfully identified, managed, mitigated and communicated (Gille et al., 2020; Akyüz et al., 2021). Despite the aspiration for holistic ways of governing risks with good governance approaches, the risks are still understood primarily as those for the participants and the biobank (Akyüz et al., 2021), where the participants’ risks are often framed within informed consent process regarding their rights, interests, and moral values, e.g., risk of genomic identifiability, and mitigated accordingly in a continuously changing technical, legal and social environment (Akyüz et al., 2023). Similarly, those of the biobank are mainly technical in nature, e.g., freezing system failures or data security, which are dealt with standardized tools and international standards (ISO and IEC, 2013; ISO, 2015; 2018a). Considering that dealing with risks often cannot be limited to a well-delineated domain or expertise, good governance and effective engagement seem to go hand in hand. ELSI literature shows the need to engage stakeholders, primarily the participants, for a better governance of the biobanks (O’Doherty et al., 2011; Bromley et al., 2020; Gille et al., 2020). In this regard, including stakeholders who are not systematically considered in such engagements could benefit biobank governance, but also allow insights into the transformations and future of biobanks and biobanking.

*Risks in biobanking—Governance, assessment, and mapping*: Risk is a notion that can be defined and operationalized in a myriad of ways. According to Lupton (2024), risk can be positioned epistemologically between *naïve realism* and *“strong” constructionism* (p. 45). Our understanding of risk is marked by what stands between these two extremes, characterized by a critical thinking of risk. Rather than as something that is marked by calculability and being out there as a representation of an objective reality that is independent of the site and time, the understanding of risk in this article strives to see it as something that is nuanced, that necessitates careful consideration of the assemblages that it is intertwined with, and that is not merely technical and material but also embedded in social relations. However, “technico-scientific perspective” (Lupton, 2024) is more salient in how risks are understood and dealt with in biobanking.

The steps of dealing with risks, “identifying, controlling, moderating, and communicating,” altogether constitute the risk governance going beyond but also “integrating” the standard elements of risk analysis: assessment, management/mitigation, and communication (Jacobson et al., 2013). Risk governance is entangled with “design and role, organizational capacity, stakeholder involvement, collaborative decision making and political accountability” as well as responsibility at the institutional level while it necessitates the involvement of various stakeholders “for the development and use of scientific knowledge within the risk governance process” (Renn and Walker, 2008). Risk assessment that includes risk mapping/identification and evaluation is by nature a highly critical component for biobanking organizations (Sargsyan et al., 2020) and should be a regular practice in order to strengthen precautions and create contingency plans that must be in place for unexpected events that will affect biobanking operations (Eng and Tan, 2019).

Risks can be economic (e.g., financial sustainability), infrastructural (e.g., technical and human resources), related to participation and activity, as well as related to research community and should always be approached with holistic thinking so that they can be managed successfully (Akyüz et al., 2021; Rychnovská, 2021). Therefore, in order to achieve effective risk governance, biobanks should implement an adaptive governance model that is able to adjust to the technoscientific and infrastructural developments as well as legal changes and emerging ethical/societal concerns (Akyüz et al., 2021).

There are numerous methods and tools available for supporting an organization to identify, map, assess, and manage risks (ISO, 2018b; 2019). Selecting a tool is directly linked to how it is going to be used and the decisions that are needed to be made. Such tools include the following: Political, Economic, Social, Technological, Legal, and Environmental factors (PESTLE) for institutional risk assessment that takes into account major factors surrounding the institution (Aguilar, 1967; Narayanan and Fahey, 1994; Schmieder-Ramirez and Mallette, 2015), Fishbone analysis (Ishikawa diagram) for cause and effect analysis (Ishikawa, 1986), Failure Mode and Effects Analysis (FMEA) for quantification of risk and severity of failures in a process (International Electrotechnical Commission, 2018), Strengths Weaknesses Opportunities Threats (SWOT) for long-term/strategic planning (Puyt et al., 2023), and, risk matrix (Baybutt, 2015; Elmontsri, 2021) among others. Considering the variation among such tools, a situation may necessitate multiple tools being used at the same time for effective assessment and management of risks.

Risk assessments can be implemented as part of biobanking standardization and business continuity or sustainability (Parry-Jones et al., 2016) but also as part of the existing legal and ethical requirements for risk assessment in biobanking, as it is found in several instruments. The European Union (EU) General Data Protection Regulation 2016/679 (GDPR), for instance, has a European impact and global reach, requiring to conduct a Data Protection Impact Assessment (DPIA) prior to the start of personal data processing (Article 35) (European Parliament and Council of the European Union, 2016). Consequently, risk assessment regarding data protection cannot be considered a voluntary practice that biobankers may or may not choose to adopt, it is rather a legal requirement. Furthermore, institutions such as funding agencies can request compliance with additional ethical requirements and soft laws as a precondition to granting funds, for instance as part of the European Union’s Horizon Europe funding scheme.

In this paper, we build on stakeholder engagement and qualitative social science methods to move the focus among a diverse set of stakeholders to a key actor: the biobankers, understood broadly as biobank staff. We consider biobank staff as central to the functioning of the biobank and this shift in focus avoids seeing the biobank primarily and merely as a storage facility. The site of this study is a newly founded biobank in Cyprus that has a well-documented governance structure (Akyuz and Mayrhofer, 2021), a continuous development of standardized ways of dealing with risks and quality management including efforts towards adopting ISO standards (ISO9001:2015 awarded; ISO15189 and ISO20387 work ongoing), as well as being under external review through a funding agency that provided the majority of the funding. These factors, as well as the risk governance practices that will be discussed further, make the biobank a suitable site for the research at hand. With this research, we include the biobankers in the mapping of the risks with the case of biobank. cy and in so doing, we open the discussion not only to *what the risks are* from the situatedness of varying roles and expertise within a biobank, but also to *what a biobank is*. In this regard, by analyzing an engagement process with the biobankers and setting a distinct example for risk mapping, we instrumentalize the identification of risks to understand the emerging structure of biobanks. This specific case brings with it new insights regarding central issues to biobanking efforts, such as sustainability and governance.

## 2 Materials and methods

Stakeholder engagement and strategies are key topics in the development of biobanking. An extensive body of ELSI literature has highlighted the importance of including publics and patients in the biobanking discourse (BBMRI, 2013; Mitchell et al., 2015) and developed approaches such as consensus conferences and citizen panels to explore their opinions, attitudes, and knowledges (Burgess, 2014; Goisauf and Durnová, 2018). However, there is less attention on biobankers, i.e., those people who are involved in the biobanking process, regarding their needs and opinions on biobanking practice (Goisauf et al., 2019) and in regard to risks in biobanking (Akyüz et al., 2021).

Social science methodology, especially from Science and Technology Studies (STS), has provided approaches to explore the situated knowledges and experiences of various stakeholders in connection to a specific practice. The methodology developed for risk mapping particularly builds on and integrates tools and concepts mainly from Situational Analysis (SA) (Clarke, 2005; Clarke et al., 2015, 2018), but also from the card-based discussion method IMAGINE (Felt et al., 2014; Felt et al., 2017; Felt et al., 2018) and the ECOUTER engagement process (Murtagh et al., 2017; Wilson et al., 2017). To our knowledge, these tools have not been used in scholarly literature for risk mapping, but rather mainly served as qualitative research methods. The strengths of these methods are the use of powerful visualizations, such as maps or cards, and the creation of an interactive space to generate knowledge from practical experience as a process.

### 2.1 Case description and sample

*biobank.cy*: The mapping exercise that will be explained in detail in the next section was conducted at biobank.cy, an institution that strives to become the pan-Cypriot biobank for health research. Through the European science and technology funding program (Horizon 2020 Teaming), a long-term project called *CY*-Biobank has facilitated the development of biobank.cy as a Center of Excellence in Biobanking and Biomedical Research. Although the biobank itself has a history that goes back to 2011 as the first biobank in Cyprus under the Molecular Medicine Research Center (MMRC)/University of Cyprus (UCY), with the named grant, it has been possible to create a strategic partnership with renowned institutions (namely, BBMRI-ERIC—a European research infrastructure for biobanking and the Medical University of Graz and RTD Talos, a small-medium enterprise in Cyprus) in line with the aims of the funding scheme to allow transfer of knowledge and expertise. This meant on the one hand, the implementation of state of the art technological and infrastructural dimensions of biobanking, but also increased attention to responsible research and innovation and public engagement among others, putting in place a good governance.

To contextualize further, the Center of Excellence consists of five pillars: the Biobank, the Molecular Medicine Research Center, the Diagnostic Lab, the Education Hub, and the Innovation Hub. All activities are aimed at collecting, analyzing, and preserving biological samples and health data in a state-of-the-art biobank and utilizing them for scientific, diagnostic, and educational innovation, specifically, to create new knowledge for improving human health and contribute to the prevention, diagnosis, prognosis, and therapy of diseases. In this regard, while biobank.cy is part of an academic institution, the biobank is not directly linked to any hospital. The Center of Excellence, however, works with several private and public institutions, and it employs personnel from different disciplines with a variety of expertise, e.g., nurses, biobank technicians, researchers, experts in IT and ethical, legal and societal issues.

Based on the understanding that risk management should be an ongoing, adaptive process for an organization where risks are reviewed and updated regularly, and in line with the highlighted diversity of the personnel, a risk mapping exercise was organized with biobank/*CY*-Biobank project staff, led by BBMRI-ERIC social scientists leading to group discussions and maps that form the empirical material for the analysis in this paper. This exercise has been conducted as a case study rather than as part of the regular risk assessment processes of the biobank. Considering that one of the main challenges for biobanks undertaking risk assessment is to identify the different types of risks, biobank. cy had already conducted four types of analysis within the context of annual reviews. These are PESTLE, SWOT, FMEA, and DPIA analyses that were prepared as part of the biobank’s business plan, in the process of ISO certification/accreditation and for GDPR compliance.

The empirical material for the risk mapping was generated during a 2-days’ workshop with the staff of biobank.cy Center of Excellence (*n* = 20) conducted on-site in Cyprus in June 2022 along with four social scientists as co-organizers/moderators. The participants of the workshop reflect the variety of roles, backgrounds, and hierarchical positions in the biobank; positions represented include technicians, nurses, managers and researchers, including in ethical/legal issues. Before the start of the workshop, the external participants of the workshop (a bioethicist involved with the biobank and the research team comprising of four social scientists), were invited to a tour of the biobank led by some of the staff members. The discussions in the workshop were audio-recorded and the produced anonymous maps and typologies have been scanned.

### 2.2 Ethics

All data was collected based on written informed consent of the participants, following explanation to the participants the aims, scope and methods of the research including the use of audio recorders during the group discussions. Voluntary participation was ensured by communicating to the participants their rights to privacy, confidentiality as well as right to withdrawal without any justification at any time. Data was collected within the scope of an ongoing international project (*CY*-Biobank) and included only participants from the research consortium itself. The research involved neither vulnerable groups nor individual-level data collection nor sensitive personal data; participants were neither subject to any treatment nor were they required to behave in a certain way and their participation did not involve any physical or psychological risks. The collected data was kept confidential and were anonymized by the researchers who had designed and executed the group discussions. In line with the measures of privacy, no direct quotation from individuals that may make them identifiable has been shared or included in this article.

### 2.3 Conducting risk mapping as interactive engagement exercise

**FIGURE 1:**
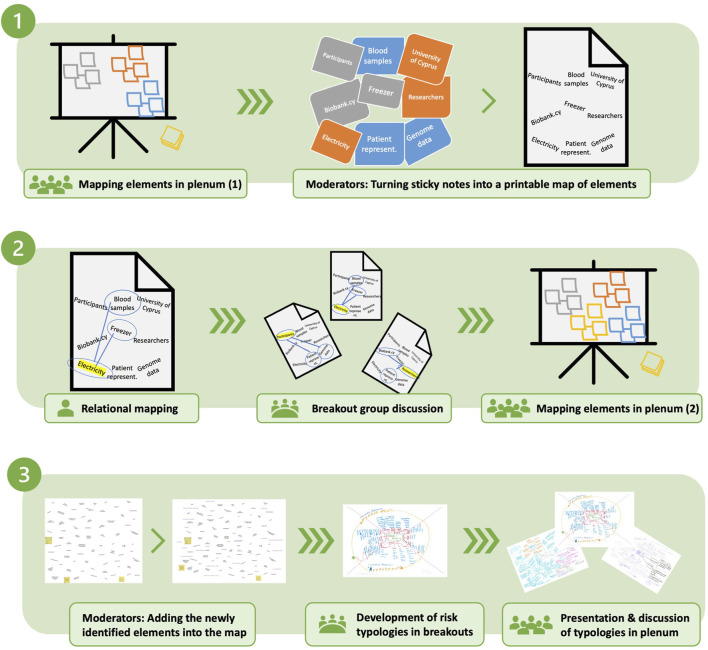
Overview of the risk mapping exercise, highlighting the major steps.

The methodology incorporates brainstorming in plenum, solitary exercises, moderated group discussions and exercises, presentations, and plenum discussions, where the materiality of working with sticky notes, creating and using maps and typologies have contributed to hands-on and active participation from all attendees. The mapping exercise took place in three consecutive steps as follows (Figure 1).

#### 2.3.1 Step 1: mapping elements in and of the biobank

In the first step, “situational mapping” from SA was used as an activating exercise to set the “situation” of biobank.cy. Participants were asked to produce sticky notes to identify elements *in* and *of* the biobank (“elements” broadly includes tasks, materials, values, issues, etc.). The moderator collected the sticky notes on a wall and moderated the plenum discussion to encourage continued participation and the generation of new elements while the discussion was ongoing. The outcome was a “situational map” of 73 elements that constitute biobank. cy.

#### 2.3.2 Step 2: building relations

The “situational map” was transcribed and printed on papers to continue with “relational mapping,” which is a systematic way of identifying relationships between elements that may not necessarily be put in relation with each other (Clarke, 2005). Clarke suggests this to be done by hand by centering on an element in a map of elements in a situation and drawing a line to each other element one by one in order to “*specify the nature of the relationship by describing the nature of that line*” (p. 102, italics in original). Considering that relational mapping is used in situational analysis for “creativity” and to make visible what is not immediately visible, it was used in this context towards similar aims as follows. Organized into breakout groups of on average six participants and one moderator, each participant received a copy of the map of elements and was asked to choose one element and draw lines to other elements for which they see a strong relationship. This solitary relational mapping was followed by a moderated discussion of identified relationships within the breakout group. A representative from each breakout group presented key discussion points in plenum followed by an open discussion. During this discussion, the participants were asked to expand the map with new elements (*n* = 29) resulting in a saturated “situational map” (*n* = 102).

#### 2.3.3 Step 3: producing risk typologies

Following the situational mapping, the participants were placed in the same moderated breakout groups and distributed the updated situational maps. After a short presentation of a generic typology of risks in biobanking (Akyüz et al., 2021) and equipped with their understanding of the “situation,” the participants were asked to produce a biobank.cy-specific typology of risks building on the biobank’s “situation” within the group. Here, the research team utilized the mapping approach from ECOUTER (Murtagh et al., 2017). Following the completion of the typologies by each group, representatives were asked to present their typologies in plenum in the final discussion which resulted in situated typologies of biobanking risks.

### 2.4 Analysis

The recordings of all discussions, plenum, and breakout groups, were interpreted by the research team consisting of the social scientists in multiple data analysis sessions. In a first step, coding strategies from Grounded Theory methodology (Charmaz, 2014; Clarke et al., 2018) were used to break up segments of the transcripts to capture and interpret its meaning. In a second step, key constitutive topics were identified and formulated as concepts. Hence, the concepts were inductively developed from the empirical data to describe the “situation” of biobank.cy and the respective assessment of risks. For instance, the practices described in the discussion illustrate not only biobanking routines on a mere technical level, but allow altogether and in relation to institutional, political, and cultural contexts, to also explore more abstract but important aspects such as trust. While the maps generated during the workshop were mainly used as tools in the discussion to support the participants in exploring the situation and to reflect their practices, we used a superimposed version of the relational maps to visualize strong associations across the discussion groups to enable a chance of perspective at a later stage of the interpretation process to discover further constitutive relations characterizing the “situation” biobank.cy.

## 3 Findings

**FIGURE 2:**
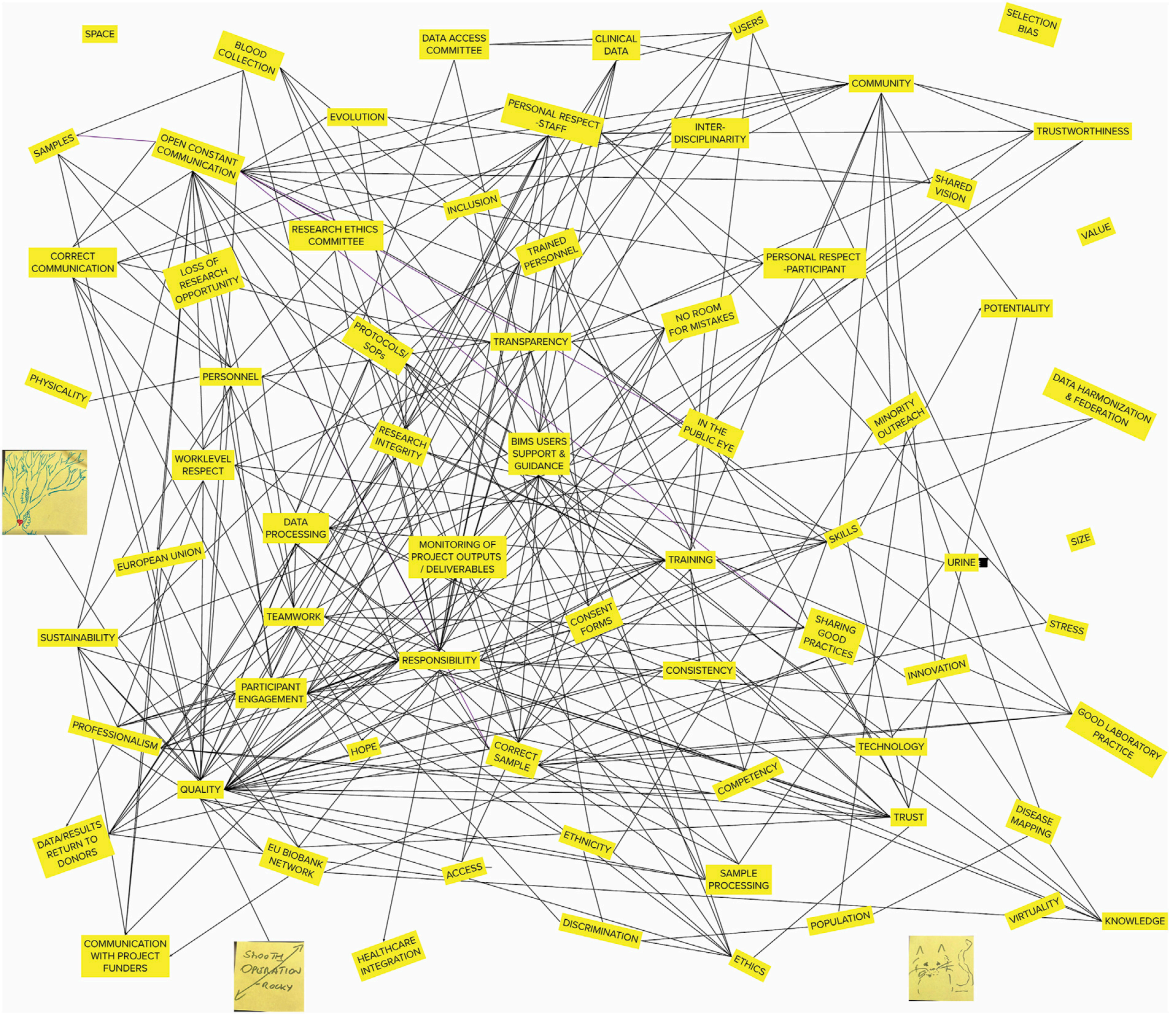
Superimposed relational map of all participants. Writings and highlights of single elements have been excluded for simplicity.

The analyzed group and plenum discussions as well as the produced maps and typologies revealed that topics that are taken to be central in risk assessment and mitigation in biobanks were not the only dominant issues raised by the biobank personnel (see Figure 2 for a superimposed relational map). Due to the risks of re-identifiability for the participants, direct quotations from the empirical material are not provided to protect their anonymity.

While topics like collection and sharing of samples and data, ethics, the healthcare context, knowledge production were not entirely absent, they were often discussed within the social dynamics of the biobank, where participants’ frequent reference to the importance of human relations and interdependencies within the team suggest the notion of the biobank imagined as a living organism, to the extent of literal description of one with a “heart in the middle.” The metaphor of an organism is an outcome of the interpretative analysis to illustrate the dynamics and social relations beyond rather static definitions of biobanks as repositories.

The use of the term organism as a focus within social enquiry has been foundational since classical sociologists such as Comte, Spencer and Durkheim used it (with nuanced differences) at the turn of the previous century (Levine, 1995). More recent is its use, for instance in the form of “superorganism” to understand epistemic cultures as in the case of a comparison of high energy physics and molecular biology laboratories (Knorr-Cetina, 1995, 1999). Rather than following oft-used infrastructure concept to study similar largescale technical organizations (Star and Ruhleder, 1996; Larkin, 2013; Slota and Bowker, 2017) or applying the existing organism/superorganism conceptualizations, we will make use of *organism* as a metaphor for the biobank to critically think about risks in biobanking through a specific case, not only due to its usefulness to make sense of the situation at hand, but also as it has been an outcome of the interpretative analysis. Thus, the findings will be framed through the metaphor of a *growing*, *living* organism *within an environment*.

### 3.1 Biobank as a growing organism: building up not just the samples and data, but a team

The “organism” metaphor connotes on the one hand the temporal dimension of change and spatial organization. Temporally, the biobank as a whole invests much of the energy to “building up” an institution, infrastructure, and organization, where being able to produce “biovalue” (Mitchell and Waldby, 2009; Birch and Tyfield, 2012; Birch, 2016) and proving this to the external stakeholders is a fundamental goal. Just like an organism grows, the growth of the biobank corresponds to the development of the parts that are making up the organism. While biobank could be considered as a technical thing with the imagery of computers, −80°C freezers or liquid nitrogen tanks where samples and data are the major elements, the discussions with the biobank team revealed that the growth of the individuals working within the biobank team are as noteworthy as the samples and data. In this regard, development of individual’s technical and professional capacity as a person, who is assigned certain tasks and responsibilities, was discussed as central for the successful growth of the institution in terms of assessing and mitigating risks.

Considering the spatial components, a biobank is greater than the sum of its parts, and certainly goes beyond the physical setting and instrumentation. Against the expectation that objects and practices like the standard operating procedures (SOPs) would be the primary point of reference in discussion of risks during the data gathering process, the individual-group dynamics, e.g., capacity to cooperate, was consistently at the core. Concretely, rather than merely following guidelines or protocols, the individuals described how they were expected to “learn to learn,” even practices like troubleshooting. This was on the one hand justified by emphasizing the differences of each individual and their capacities. On the other hand, it related to the interdependency that the participants felt, culminating in discussion of trust, respect and confidence in oneself and others. In this sense, what started as discussion of risks often wandered towards topics of effective communication, building skills and support as well as transparency within the institutional walls and beyond, moving from the risks emanating from an individual’s work to strengthening the mitigative capacity as a team, which will be discussed further in the next section.

Just as growing is a fragile state of being, the “building up” phase for a biobank is also a struggle, especially in identifying the contribution of the biobank to broader community and self-positioning as well as learning how to sustain relevant resources, from funding to qualified personnel, trust to samples and data. At the core of the ‘start-up’ talk is often the effort of making value out of biological materials and producing value with them that the other biobanks cannot. Here, an obvious limiting factor identified in the discussions, as with many biobanks, was the difficulty of sustaining the steady flow of samples and data that the built infrastructure has the capacity for, and funders are expecting. In this regard, while acknowledging the value of communication with potential participants and building trust, the situatedness of the biobank.cy as an institution, such as not being within a research hospital, was also framed relevant to make sense of the success of communicative practices in terms of recruitment of participants. Within this temporal stage of growth, along with workflows, lifecycles, quality aspects, participant and volunteer engagements that are also relevant, the discussions often led to participants highlighting the stress at the individual and team level due to the need to keep an eye on everything since growing means change, especially considering the changing environment the biobank is located in.

### 3.2 Biobank as a living organism: social dynamics and trust

One definition of organism is “[a] whole with interdependent parts” (Oxford English Dictionary, 2022b) and this interdependency in the case of a biobank takes the focus from mere technical perspective to a holistic one where the social is at the core. One key concept that classical sociologists shared when using organismic analogies as heuristic devices to study society was the shift of focus from the individual to understanding their relationships with and within larger social structures (organizations/institutions). Focus on levels of cooperation and strategies to regulate conflict were at the core of their theorizing on what makes it possible for society to exist in a stable way as mentioned above. Extending this metaphor of a social organism to the exploration of a biobank brings the importance of social dynamics, trust and risk to the fore.

While each individual is assigned specific roles and tasks, the biobank becomes and acts as an actor through the daily interactions and strategies of individuals within. Viewed in this way, the technicalities of dealing with everyday situations, e.g., limiting permissions, are not entirely capable of preventing risks such as mistakes. This highlights the previously mentioned aspects of trust, confidence and respect within the team. Beyond “risks associated with human resources, such as the human error” (Barcan et al., 2020), individuals as social actors are often overlooked in the literature on risks in biobanking, but played a central role in our data-gathering discussions, such as through mutual respect, responsibility and learning by examples.

Trust serves as the key concept that is inseparable from the social dynamics, but it is not merely about the social, and goes beyond touching on aspects such as data, quality, participants, the publics, external researchers or the funders. Lack/loss of trust is clearly a risk from multiple aspects and is countered with discussions of confidence. While standardization and optimization of procedures, workflows, samples and materials were discussed, they were often instrumental to the discussions of how the team comes together and is sustained in the long run. In other words, our data indicate that the mitigation of risks related to technical aspects are handled in a more straightforward manner. On the contrary, becoming a team cannot be simply implemented according to guidelines or frameworks, but is built through social exchanges, building confidence and (self-)reassurance of the capacity to cope with issues as a team. The metaphor of the “heart in the center” of the biobank, both raised as a discussion point in a brainstorming session and represented by a participant in a sticky note as a tree with a heart symbol (included in the situational map at the left hand-side), is also about care that starts at the individual level with responsibility and neatly tied to mutual trust. As noted in a previous typology of risks, risks in biobanking are entangled and often feature multiplicity necessitating adaptive risk governance and exchanges between units in a biobank as well as with stakeholders for thorough assessment (Akyüz et al., 2021). Here, with the focus on trust, the biobank personnel’s discussion adds a further layer to the entanglements that relate to social dynamics in day-to-day workings of the infrastructure.

### 3.3 Biobank as an organism within an environment: values and situatedness

Just as an organism is immersed within an environment that it is living, biobanks are also situated in multiple ways: geography, politics, culture, funding, physical setting among others. Considering that neither the environment nor the biobank is static but often in a kind of state of maintenance or homoeostasis, a concept defined as “maintenance of a dynamically stable state within a system by means of internal regulatory processes that tend to counteract any disturbance of the stability by external forces or influences; the state of stability so maintained” (Oxford English Dictionary, 2022a), the risks that relate to the outside were often discussed from an internal-external perspective. As noted earlier, the growth dynamic was associated with stress at the individual and team level; however, to put this succinctly in context, this is on the one hand related to the aim to build an infrastructure that the team can passionately support internally and is justified and convincing for the external stakeholders to interact with. On the other hand, the values that are clearly upheld in the discussion, such as “transparency” indicate that both the individuals and the team are continuously aware of their position and responsibility to prevent major mistakes. This, however, is not independent of discussions of value and valuation in recognition of the importance of the social license to biobank, from societal support and participation to funding. In this sense, value making is not restricted to producing knowledge out of samples and data and being embedded in a tissue economy (Waldby and Mitchell, 2006; Mitchell and Waldby, 2009), where building the biobank itself as an exemplary effort is part of the value-making process. This means achieving excellence and communicating to the multiple stakeholders the “biobanking” success but at the same time keeping up with the changing expectations of the research community as well as the routine of relying on a favourable environment and shaping it at the same time. Biobanks need solid ground and the discussions highlighted how internal and external environments should both be considered in assessing risks.

## 4 Discussion

In imagery of biobanks, people are often missing, and if they are portrayed, they are habitually partially included in the form of the arms of a person wearing a lab coat, with glove-covered hands holding a pipette and a sample against a background of further samples, freezers and liquid nitrogen tanks. At least two things in evolving biobanking infrastructures are not fitting this picture: first, biobanks are more and more understood as data infrastructures as much as they are physically housing samples (question: where are the data?). Second, biobanks are lively, with actors that are often (made) invisible: biobank staff (question: where are the humans?). In this paper, based on a group discussion and mapping-focused methodology, the discussion of risks converged in multiple ways on what a biobank is.

This research started with the critical observation that biobankers/biobank staff are understudied as social actors, especially in the context of their (potential) contribution to risk governance. Furthermore, a biobank is a *living*, *growing* organism in a *changing environment*, in need of adapting. Acknowledging and engaging biobank staff directly as a key stakeholder in risk mapping allowed bringing to the fore the taken-for-granted aspects, such as team building, social dynamics and trust, values and situatedness. Within this organismic view of biobanks, *trust* and *sustainability* emerge as cross-cutting elements, culminating in the crystallization of an *adaptive risk governance*.

The findings reveal that trust is a ubiquitous element not only as it relates to patients, funders or the informed consent process but also as an element that keeps the biobank together highlighting the interdependency. Trust is a widely studied concept. Scholars have noted that “modernity is largely structured by trust vested in abstract systems which by its very nature is filtered by the trustworthiness of established expertise” (Giddens, 1990) and the recent COVID-19 pandemic has shown the need for a “cosmopolitan force” (Beck, 2009) highlighting the link between epistemic and political authority in a global setting from data sharing to transnational cooperation (Hurlbut, 2017). The interdependency, both in a global setting and at an institution, intersects with risks in such ways that the breakdown of the communication, relations and trust may consequently lead to a breakdown of the processes, e.g., regarding data. Indeed, we suggest that the success of capturing the diversity of risks is possible by considering the interactions and the social aspects of the involved work, not merely between the institution and the outside, but also within.

Going from a global setting of managing risks to the setting of the biobank, trust and reliance on expertise of each other becomes more relevant. In science, sites of interactions such as “trading zones” (Galison, 1999) and interactions among colleagues from close but different specialties such as between theoretical and experimental physics (Reyes-Galindo, 2014) or bioinformatics and wet lab in life sciences (Penders et al., 2008) have centered around trust and trust relations. This is especially relevant considering the ‘tacit knowledge’ that individuals possess specifically in the laboratory/technical setting (Collins, 1974). Consider as examples the processes from DNA extraction to data protection: there is a variety of “epistemic cultures” enmeshed with day-to-day practices (Knorr-Cetina, 1999) such as among IT or quality management experts and nurses at a biobank. Furthermore, various forms of expertise from a “relational” perspective (Irwin and Wynne, 1996) are involved, such as ways of communicating with research participants while taking informed consent or persuading funders for continued support or engaging with variety of publics. The organismic metaphor allows to extend this literature by highlighting how fragility and strength are both cut across by trust: The biobank staff need to trust the competence and ethical standards of each other in different domains of expertise leaving room for individualization while not losing the cohesion at the institution and the confidence in it, especially in dealing with risks.

Focus on risk governance allows to concentrate on sustainability of practices around risks. Similarly, the organismic metaphor brings change and adaptation to the center stage. Practices such as DPIA, align with the metaphor of a living and changing thing. To exemplify, a DPIA is imagined to be a living document rather than a consequence of a one-off process and is expected to remain relevant over time, being updated according to the changes within and outside of the biobank. While a DPIA is an exemplary process that is part of the European legal landscape, similar arguments can be made for ethics assessments, which should go beyond being a mere check boxing, but ideally become embedded in practices. Tools such as DPIAs or ethics self-assessment surveys bear the potential of being mistaken as (sole) risk assessment practices; on the contrary, the organismic metaphor of a biobank allows to expand the repertoire of identifiable risks, contributing to risk governance. Thus, rather than putting weight on specific actions undertaken by specific individuals at the biobank, organismic metaphor allows a more sustainable approach that suggests involving diverse set of people for incorporating risk-conscious practices into the individuals’ routine.

Scholars have previously come up with typological categorization of risks in biobanking arguing for a holistic and adaptive risk governance rather than merely following templates of already identified risks, based on the observation that risks are entangled, having both downstream/upstream and multiplicity (Akyüz et al., 2021). In this paper, we have focused on a specific exercise of risk mapping through such a holistic approach, by bringing together a group of biobank staff with slightly diverse but related expertise. We argue that such a risk mapping practice not only contributes to better risk governance, but also can be regarded as a key step in making risks governance adaptive.

Adaptive character can be realized through two aspects. First, as discussed with the centrality of trust, the interdependencies and diversity can be turned into an asset through risk mapping by biobank staff. In this regard, risk governance becomes both more inclusive by bringing in an often forgotten but central stakeholder, the biobank staff, and also being reflexive as the custodians who are the closest to samples and data are able to bring their perspectives into the mapping. Second, the situatedness of the biobank is represented through the systematized focus on risks via the mapping exercise of risks by a representative group of biobank staff. Just as the organismic metaphor stresses the change within and in the environment, being up-to-date against a continuous change is central. The situatedness of the biobank and the risks mapped belong to a specific timepoint necessitating the continuation of such practices as part of the sustainability of risk governance. In this regard, the adaptive character is in line with the idea of the biobank itself in terms of being a major shift from the concept of collection and use of samples/data for one-off specific research projects by specific researchers, to the collection and storage of samples and data for future use in research projects that are in line with ethical and legal requirements, in ways that may as yet be unforeseen from a current perspective.

## 5 Conclusion

Adaptive risk governance is a future-oriented effort that takes uncertainty seriously. The biobank relies on a diversity of expertise that is at hand in-house and in the risk mapping practice this internal capacity was made use of, unlike similar other components of good governance for “future-proofing” such as “expert advice, compliance, external review and partnership” (Gille et al., 2020) that rely on external expert support. The organismic metaphor and the three facets of the biobank as a growing, living organism within a changing environment reveal how the cross-cutting elements of trust and sustainability can be operationalized through better involvement of the key stakeholders, among them the often-overlooked biobank staff. Focusing on the situatedness of the dynamics within biobanks, as a central infrastructure for improving public health, and their activities highlights the importance of prioritizing relations at the core of risk governance and promoting ethicality in the biobanking process. Systematically including stakeholders who are often not prioritized in engagement activities could benefit biobank governance, especially from a risk-focused perspective, but also allow a better understanding of the evolution biobanks as infrastructures and biobanking as practice.

## Data Availability

The original contributions presented in the study are included in the article/supplementary material, further inquiries can be directed to the corresponding author.

## Abbreviations

BBMRI-ERIC, Biobanking and Biomolecular Research Infrastructure—European Research Infrastructure Consortium; DPIA, Data Protection Impact Assessment; ELSI, Ethical, legal and societal issues; EU, European Union; FMEA, Failure Mode and Effects Analysis; GDPR, General Data Protection Regulation; IEC, International Electrotechnical Commission; ISO, International Organization for Standardization; IT, Information Technology; MMRC, Molecular Medicine Research Center; OECD, Organisation for Economic Co-operation and Development; PESTLE, Political, Economic, Social, Technological, Legal, and Environmental factors; SA, Situational Analysis; SOPs, Standard Operating Procedures; STS, Science and Technology Studies; SWOT, Strengths Weaknesses Opportunities Threats; UCY, University of Cyprus; UNESCO, United Nations Educational, Scientific and Cultural Organization.
